# Long Term Prognostic Impact of Sex-specific Longitudinal Changes in Blood Pressure. The EPIC-Norfolk Prospective Population Cohort Study

**DOI:** 10.1093/eurjpc/zwab104

**Published:** 2021-07-05

**Authors:** Tiberiu A Pana, Robert N Luben, Mamas A Mamas, John F Potter, Nicholas J Wareham, Kay-Tee Khaw, Phyo K Myint

**Affiliations:** 1 Aberdeen Diabetes and Cardiovascular Centre, School of Medicine, Medical Sciences & Nutrition, University of Aberdeen, Room 4:013, Polwarth Building, Foresterhill, Aberdeen AB25 2ZD, UK; 2 Ageing Clinical and Experimental Research (ACER) Team, University of Aberdeen, Aberdeen, UK; 3 Clinical Gerontology Unit, Department of Public Health & Primary Care, University of Cambridge, Worts Causeway, Cambridge CB1 8RN, UK; 4 Keele Cardiovascular Research Group, Keele University, Stoke-on-Trent, University Road, Staffordshire ST5 5BG, UK; 5 Department of Clinical Neuroscience, Norfolk and Norwich University Hospital, Norwich, UK; 6 Norwich Cardiovascular Research Group, Norwich Medical School, University of East Anglia, Norwich Research Park, Centrum, Colney Lane, Norwich NR4 7UG, UK; 7 MRC Epidemiology Unit, Institute of Metabolic Science, University of Cambridge School of Clinical Medicine, Cambridge CB2 0SL, UK

**Keywords:** Blood pressure, Longitudinal, Trajectory, Sex-specific, Mortality, Cardiovascular disease

## Abstract

**Aims:**

We aimed to determine the sex differences in longitudinal systolic and diastolic blood pressure (SBP and DBP) trajectories in mid-life and delineate the associations between these and mortality (all-cause, cardiovascular, and non-cardiovascular) and incident cardiovascular disease (CVD) in old age.

**Methods and results:**

Participants were selected from the European Prospective Investigation into Cancer, Norfolk (EPIC-Norfolk) cohort study. Sex-specific trajectories were determined using group-based trajectory models using three clinic BP measurements acquired between 1993 and 2012 (mean exposure ∼12.9 years). Multivariable Cox regressions determined the associations between trajectories and incident outcomes over the follow-up (median follow-up 9.4 years). A total of 2897 men (M) and 3819 women (F) were included. At baseline, women were younger (F-55.5, M-57.1), had a worse cardiometabolic profile and were less likely to receive primary CVD prevention including antihypertensive treatment (F-36.0%, M-42.0%). Over the exposure period, women had lower SBP trajectories while men exhibited more pronounced SBP decreases over this period. Over the follow-up period, women had lower mortality (F-11.9%, M-20.5%) and CVD incidence (F-19.8%, M-29.6%). Compared to optimal SBP (≤120 mmHg) and DBP (≤70 mmHg) trajectories, hypertensive trajectories were associated with increased mortality and incident CVD in both men and women during follow-up at univariable level. These associations were nevertheless not maintained upon extensive confounder adjustment including antihypertensive therapies.

**Conclusion:**

We report sex disparities in CVD prevention which may relate to worse cardiometabolic profiles and less pronounced longitudinal SBP decreases in women. Effective anti-hypertensivetherapy may offset the adverse outcomes associated with prolonged exposure to high blood pressure.

## Graphical abstract

**Figure:**
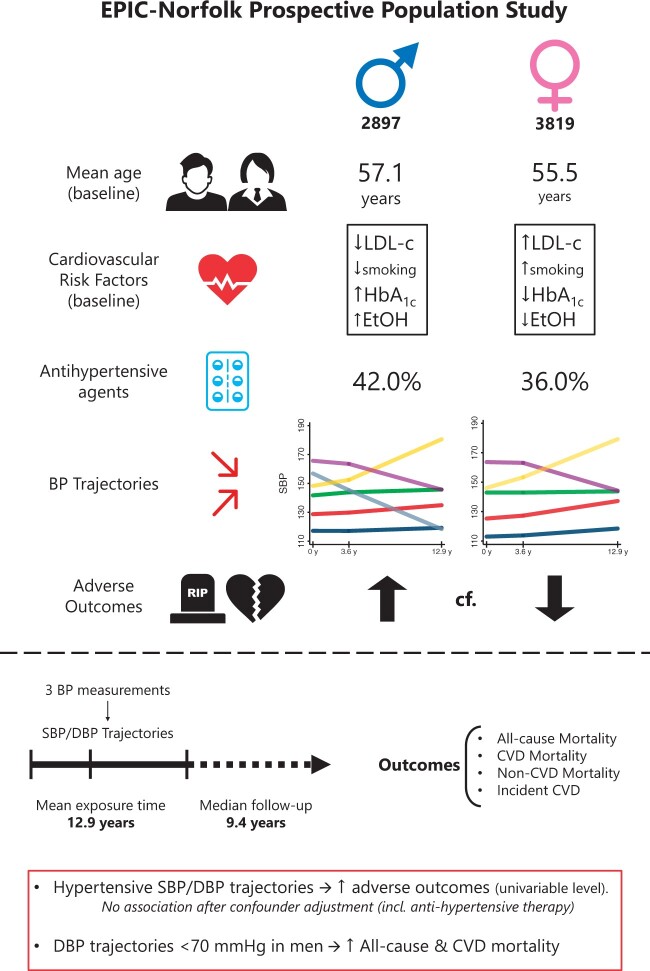

## Introduction

While the magnitude of blood pressure (BP) elevation predicts the strength of association between hypertension and adverse outcomes,^1–3^ long-term exposure to elevated BP values is also important. Long-term BP trajectories allow additional factors including antihypertensive treatment and ageing-related changes in arterial stiffness and BP^4^ to be considered. Group-based trajectory modelling (GBTM) is a data-driven approach which allows the derivation of clusters of individuals exhibiting statistically similar longitudinal trajectories of a given parameter, such as BP.^5^ Unlike approaches which define longitudinal trajectories *a priori*, GBTM does not rely on assumptions based on subjective and inflexible *ex ante* assignment rules.^6^ GBTM therefore enables the identification of new and previously unrecognized longitudinal trajectories.^6^ In this data driven approach, the clusters thus identified do not represent distinct fixed entities but rather convenient groupings of individuals following similar trajectories. Individuals are assigned to each trajectory group based on a probability of group membership and therefore the interpretation of such trajectories depends on these considerations.^5^^,^^6^

Hypertensive trajectories are associated with an increased risk of incident stroke,^7^ renal disease,^8^ cardiovascular disease (CVD),^9–11^ and mortality.^12–14^ Nevertheless, significant sex differences have been demonstrated in BP trajectories.^15^ These may also mediate sex differences in CVD epidemiology, given recent findings of such differences in the relationship between longitudinal BP trajectories and incident atrial fibrillation (AF).^11^ Nevertheless, no studies have previously evaluated the associations between sex-specific longitudinal BP trajectories and mortality and overall incident CVD. Furthermore, current hypertension guidelines lack sex-specific recommendations.^1–3^

In this study, we aimed to determine the sex differences in longitudinal systolic BP (SBP) and diastolic BP (DBP) trajectories in mid-life and delineate the associations between these and mortality (all-cause, cardiovascular, and non-cardiovascular) and incident CVD in older age using data from the European Prospective Investigation into Cancer—Norfolk Cohort (EPIC-Norfolk).

## Methods

### Ethical considerations

This study was conducted in accordance with the Declaration of Helsinki (1975) and later amendments. Ethical approval was obtained from the Norwich Ethics Committee. All participants gave informed signed consent for the examination of medical records and use of the data. The data that support the findings of this study are available from the corresponding author, upon reasonable request.

### Data source

Participants were selected from the European Prospective Investigation into Cancer, Norfolk (EPIC-Norfolk) prospective cohort study. Study recruitment methods have been previously described.^16^ In brief, men and women aged 40–79 (at study baseline) from 35 General Practices in Norfolk, England were invited to participate. Three health checks (HCs) occurred between 1993–1998 (study baseline), 1998–2000, and 2004–2012. At each HC, data on age, demographic characteristics, behavioural parameters, SBP and DBP measurements, and medication were collected. Self-reported comorbidities were ascertained during the first and second HCs. A follow-up questionnaire obtained between 2000 and 2006 ascertained self-reported comorbidities before the third HC.^17^

### Inclusion and exclusion criteria

**Figure 1:**
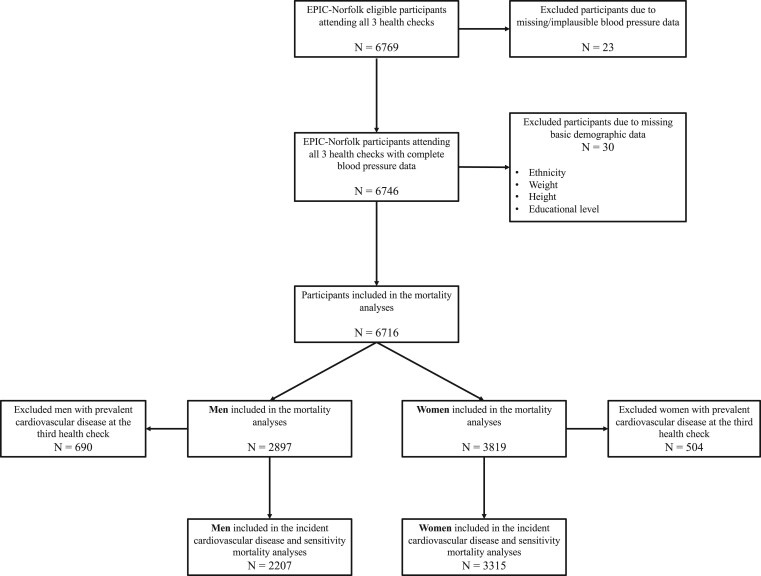
Participant population flowchart.

*Figure 1* illustrates the participant flowchart. Out of 6769 participants who attended the first three HCs of the EPIC-Norfolk study, 6716 (2897 men and 3819 women) were included in the mortality analyses, after the exclusion of 53 participants with missing or implausible blood pressure data. A total of 690 men and 504 women with prevalent CVD at the third HC (2004–2012) were further excluded from the incident CVD analyses, including 2207 men and 3315 women.

### Definition of exposure, confounders, and outcomes

#### Outcomes

All-cause mortality was ascertained using death certificate data from the Office of National Statistics.^18^ Cardiovascular mortality was ascertained using death certificate data and International Classification of Disease 10 (ICD-10) codes (I10-79) and ICD-9 codes (401–448) obtained through record linkage with the National Health Service (NHS) hospital information system and ENCORE (East Norfolk Commission Record) to allow notification of any hospital admission. Incident CVD was defined as the first date of any hospital admission/primary care diagnosis with a diagnosis comprised within the ICD-10 codes of I11-I79 and ICD-9 codes of 402-448, excluding diagnoses of essential hypertension (ICD-10: I10 and ICD-9: 401). Previously published validation studies of random samples from EPIC-Norfolk assessing the diagnoses of stroke^18^ and heart failure^19^ have shown that these parameters were ascertained with high accuracy. Furthermore, the United Kingdom National Health Service (NHS) captures almost all incident events and the EPIC-Norfolk study participants were registered with a General Practitioner and assigned an NHS number, allowing extremely robust record linkage. Therefore, the outcomes employed in our study were ascertained with high accuracy. Participants were followed up until the end of March 2018.

#### Exposures

Blood pressure measurements were acquired by a trained nurse after participants had been seated for 3 minutes in a quiet room. Two readings were taken using an aneroid Accutorr Sphygmomanometer (Datascope, UK) using an appropriately sized cuff with the participants’ arm in the horizontal position in line with the mid-sternum.^20^ The mean of the two readings was then recorded.

Longitudinal SBP and DBP trajectories across the first three HCs (mean exposure 12.9 years) were determined separately for men and women using Group-Based Trajectory Models (GBTM) and the *traj* Stata plugin.^21^ Trajectories were modelled using the censored normal distribution. The selection of the GBTM model with an optimal number of quadratic groups was informed by the Bayesian Information Criterion (BIC). The model with the least negative BIC which contained at most six distinct groups was chosen, ensuring that no group contained less than 1% of the considered population.

#### Confounders

Potential confounders considered were measured at the third HC (age, sex, ethnicity, body mass index (BMI), physical activity levels, low-density lipoprotein cholesterol, smoking status, and units of alcohol drunk) or ascertained from a follow-up questionnaire obtained before (2000–2006) the third HC (self-reported cancer, asthma, and chronic obstructive pulmonary disease). Covariates were chosen based on clinical judgement and previous literature.^7^^,^^11^^,^^12^^,^^22–24^ The estimated glomerular filtration rate was computed using the creatinine values measured at the third HC using the Modification of Diet in Renal Disease (MDRD) formula.^25^ Comorbid CVD was defined as a self-reported diagnosis of angina, myocardial infarction, cerebrovascular disease or peripheral vascular disease on/before the follow-up questionnaire or incident CVD (ICD-10: I11-I79, ICD-9: 402-448) reported during the exposure period. Co-morbid diabetes mellitus was defined as a self-reported diagnosis of diabetes reported on/before the follow-up questionnaire, glycated haemoglobin levels >6.5% (47.5 mmol/mol) or self-reported anti-diabetic medication at the third HC.

### Statistical analysis

Data were analysed using Stata 15.1 SE (StataCorp 2017, Stata Statistical Software: Release 15, College Station, TX, USA: StataCorp LLC). A 5% threshold of statistical significance was used (*P* < 0.05).

#### Missing data

Six variables collected at the third HC contained missing data. *Table 1* details the proportion of missing data for these variables. Supplementary material online, *Table S1* summarizes third HC characteristics stratified by whether data on any of these variables were missing. A total of 2364 (35.2%) participants had missing data on at least one variable. Those were significantly more likely to be younger and have higher incidence of adverse events (all-cause mortality, cardiovascular mortality, and incident CVD). Data missingness was likely dependent only on observed but not unobserved data, and subsequently missing-at-random.^26^ A multiple imputation by chained equation algorithm with 20 iterations was implemented. Variables were imputed using predictive mean matching drawing from five nearest neighbours. Age, sex, ethnicity and third HC data: weight, height, educational level, SBP, DBP, heart rate, pre-existing co-morbidities, medication, and two Nelson-Aalen cumulative hazard functions (incident all-cause and cardiovascular mortality) were used as predictors.

**Table 1:** Third health check characteristics and incident outcomes of included participants from the European Prospective Investigation in Cancer (EPIC)-Norfolk (unless otherwise stated), stratified by sex

	Men	Women	*P*-value
	2897	3819	
Age, mean (SD)			
1st health check *(1993–1998)*	57.13 (8.02)	55.54 (7.81)	**<0.001**
2nd health check *(1998–2000)*	60.75 (8.07)	59.15 (7.87)	**<0.001**
3rd health check *(2004–2012)*	70.07 (8.25)	68.47 (8.05)	**<0.001**
Ethnicity, *N* (%)			0.359
White	2883 (99.52)	3810 (99.764)	
Black	2 (0.07)	2 (0.05)	
South Asian	3 (0.10)	2 (0.05)	
Other	9 (0.31)	5 (0.13)	
Weight (kg), mean (SD)	81.19 (12.04)	68.08 (12.44)	**<0.001**
Height (cm), mean (SD)	173.50 (6.58)	160.54 (6.18)	**<0.001**
Body mass index (kg/m^2^), mean (SD)	26.95 (3.58)	26.41 (4.56)	**<0.001**
Systolic blood pressure (mmHg), mean (SD)			
1st health check	134.70 (16.28)	129.42 (16.98)	**<0.001**
2nd health check	134.66 (16.57)	129.66 (17.35)	**<0.001**
3rd health check	136.62 (15.39)	135.94 (17.15)	0.096
Diastolic blood pressure (mmHg), mean (SD)			
1st health check	83.44 (10.57)	79.12 (10.38)	**<0.001**
2nd health check	83.35 (10.70)	78.92 (10.55)	**<0.001**
3rd health check	79.40 (9.59)	77.16 (8.98)	**<0.001**
Estimated glomerular filtration rate^a^ (mL/min/1.73 m^2^), mean (SD)	73.17 (17.40)	72.44 (20.93)	0.206
Creatinine (mmol/L), mean (SD)	93.24 (20.86)	73.29 (16.56)	**<0.001**
*Missing*, *N* (%)	872 (30.10)	1184 (31.00)	0.427
HbA_1c_ (%), mean (SD)	5.84 (0.67)	5.80 (0.56)	**0.010**
*Missing*, *N* (%)	206 (7.11)	331 (8.67)	**0.020**
LDL cholesterol (mmol/L), mean (SD)	2.91 (0.97)	3.36 (0.97)	**<0.001**
*Missing*, *N* (%)	217 (7.49)	335 (8.77)	0.058
Units of alcohol drunk, median (IQR)	6.00 (1.00–12.00)	2.00 (0.00–6.00)	**<0.001**
*Missing*, *N* (%)	97 (3.35)	153 (4.01)	0.158
Educational Level, *N* (%)			**<0.001**
None	635 (21.92)	1115 (29.20)	
O-level	279 (9.63)	522 (13.67)	
A-level	1384 (47.77)	1581 (41.40)	
University degree	599 (20.68)	601 (15.734)	
Physical activity level, *N* (%)			**<0.001**
Inactive	1086 (37.49)	1381 (36.16)	
Moderately inactive	731 (25.23)	1232 (32.26)	
Moderately active	519 (17.92)	644 (16.87)	
Active	520 (17.95)	511 (13.38)	
*Missing*	41 (1.42)	51 (1.34)	
Smoking status, *N* (%)			**0.024**
Yes	90 (3.11)	159 (4.16)	
No	2762 (95.34)	3604 (94.37)	
*Missing*	45 (1.55)	56 (1.47)	
Pre-existing co-morbidities			
Cardiovascular disease, *N* (%)	881 (30.41)	817 (21.39)	**<0.001**
Diabetes mellitus, *N* (%)	315 (10.87)	265 (6.94)	**<0.001**
Cancer, *N* (%)	178 (6.14)	369 (9.66)	**<0.001**
Asthma, *N* (%)	247 (8.53)	390 (10.21)	**0.020**
Chronic obstructive pulmonary disease, *N* (%)	215 (7.42)	363 (9.51)	**0.003**
Drug therapy			
Aspirin, *N* (%)	758 (26.16)	500 (13.09)	**<0.001**
Lipid-lowering agents, *N* (%)	814 (28.1)	730 (19.11)	**<0.001**
Non-steroidal anti-inflammatory drugs, *N* (%)	908 (31.34)	794 (20.79)	**<0.001**
Anti-diabetic drugs, *N* (%)	168 (5.80)	103 (2.70)	**<0.001**
Antihypertensive agents, *N* (%)	1218 (42.04)	1375 (36.00)	**<0.001**
ACE inhibitors, *N* (%)	616 (21.26)	486 (12.73)	**<0.001**
Beta-blockers, *N* (%)	433 (14.95)	415 (10.87)	**<0.001**
Loop diuretics, *N* (%)	119 (4.11)	170 (4.45)	0.492
Other diuretics, *N* (%)	326 (11.25)	563 (14.74)	**<0.001**
Angiotensin receptor blockers, *N* (%)	174 (6.01)	264 (6.91)	0.136
Calcium channel blockers, *N* (%)	407 (14.05)	428 (11.21)	**<0.001**
Incident outcomes^b^			
Mortality, *N* (%)			
All-cause	595 (20.54)	453 (11.86)	**<0.001**
Cardiovascular	160 (5.52)	133 (3.48)	**<0.001**
Non-cardiovascular	435 (15.02)	320 (8.38)	**<0.001**
Incident cardiovascular disease,^c^*N* (%)	653 (29.59)	656 (19.79)	**<0.001**

Statistically significant results (*P *<* *0.05) are highlighted in bold.

ACE, angiotensin-converting enzyme; HbA_1c_, glycated haemoglobin; IQR, interquartile range; LDL, low-density lipoprotein; SD, standard deviation.

a Estimated glomerular filtration rate was calculated using the *Modification of Diet in Renal Disease* formula.^25^

b Incident outcomes measured during the follow-up period from the third health check (2004–2012) until the end of March 2018, resulting in a median follow-up of 9.44 years.

c Incident cardiovascular disorders reported only amongst patients without pre-existing cardiovascular disease at the third health check (*N* = 2207 men; 3315 women).

#### Descriptive statistics

Participant characteristics at the third HC were compared between men and women using the χ^2^ test, student’s *t*-test or Mann–Whitney U test as appropriate.

#### Cox regression analyses

For the mortality analyses, participants were followed-up from the third HC until either death or end of follow-up. Participants in the incident CVD analyses were followed-up from the third HC until either the incidence of a cardiovascular event, death or end of follow-up. Given that for this analysis we determined cause-specific hazard ratios,^27^ death was considered a censoring event. The median follow-up time was calculated separately for men and women using the reverse Kaplan–Meier method.

Sex-specific Cox regression models were computed for all outcomes of interest. A further sensitivity Cox regression analysis was undertaken assessing the relationship between BP trajectories and all-cause mortality only amongst participants without prevalent CVD at the third HC. The satisfaction of the proportional hazards assumption for the exposures was verified using log-negative-log plots. The BP trajectory group containing the lowest measurements was assigned as reference for all analyses. Sequentially adjusted models were constructed: Model A—Univariable; Model B—Multivariable adjustment for age and ethnicity; Model C—Model B + BMI, physical activity level, smoking and alcohol consumption; Model D—Model C + pre-existing comorbidities (CVD, diabetes mellitus, cancer, asthma, chronic obstructive pulmonary disease) and serum low-density lipoprotein cholesterol; Model E—Model D + antihypertensive treatment.

## Results

### Descriptive statistics

*Table 1* summarizes the participant sex-specific characteristics at the third HC. A total of 2897 (43.1%) men and 3819 (56.9%) women were included. Compared to men [mean age (standard deviation) = 70.07 (8.25) years], women were younger [68.47 (8.05) years]. SBP and DBP measurements were higher amongst men than women. Compared to men, women had lower rates of self-reported comorbid CVD, diabetes mellitus, cancer, asthma, and chronic obstructive pulmonary disease on or before the third HC. Women also had lower rates of self-reported therapy with aspirin, lipid-lowering agents, anti-diabetic, and anti-hypertensive agents. Over the follow-up period, men had higher rates of incident mortality (all-cause, cardiovascular, and non-cardiovascular) as well as incident CVD.

### Longitudinal blood pressure trajectories

**Figure 2:**
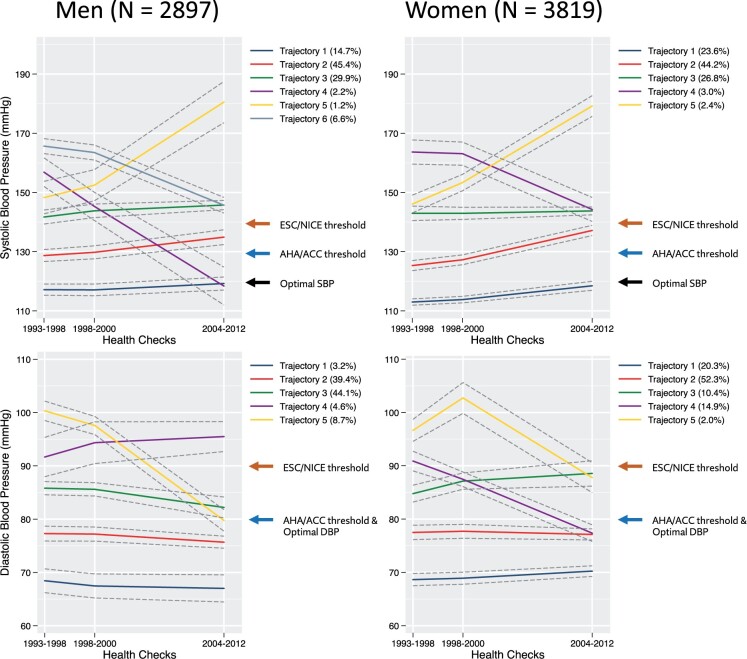
Blood pressure trajectories amongst men (*n* = 2897) and women (*n* = 3819) included in the mortality analyses. SBP trajectories in men: Trajectory 1—stable optimal SBP, Trajectory 2—stable normal/high normal SBP, Trajectory 3—stable grade 1 hypertension, Trajectory 4—well-controlled grade 1 hypertension, Trajectory 5—grade 1 → grade 2 hypertension, Trajectory 6—grade 2 → grade 1 hypertension. SBP trajectories in women: Trajectory 1—stable optimal SBP, Trajectory 2—rising normal/high normal SBP, Trajectory 3—stable grade 1 hypertension, Trajectory 4—grade 2 → grade 1 hypertension, Trajectory 5—grade 1 → grade 2 hypertension. DBP trajectories: Trajectory 1—low optimal DBP, Trajectory 2—high optimal DBP, Trajectory 3—normal/high-normal DBP, Trajectory 4—grade 1 hypertension → normal DBP, Trajectory 5—stable grade 1/grade 2 hypertension. 95% confidence intervals are represented as dashed grey lines.

There were six distinct SBP trajectories amongst men included in the mortality analyses (*Figure 2* and Supplementary material online, *Table S2*), which were characterized according to the 2018 ESC guidelines.^1^ A total of 14.7% belonged to trajectory 1 (stable optimal SBP), 45.4% to trajectory 2 (stable normal/high normal SBP), 29.9% to trajectory 3 (stable grade 1 hypertension), 2.2% to trajectory 4 (well-controlled grade 1 hypertension), 1.2% to trajectory 5 (grade 1 → grade 2 hypertension) and 6.6% to trajectory 6 (grade 2 → grade 1 hypertension). Amongst women included in the mortality analyses, five SBP trajectories were revealed: 23.6% belonged to trajectory 1 (stable optimal SBP), 44.2% to trajectory 2 (rising normal/high normal SBP), 26.8% to trajectory 3 (stable grade 1 hypertension), 3.0% to trajectory 4 (grade 2 → grade 1 hypertension), and 2.4% to trajectory 5 (grade 1 → grade 2 hypertension), respectively.

**Figure 3:**
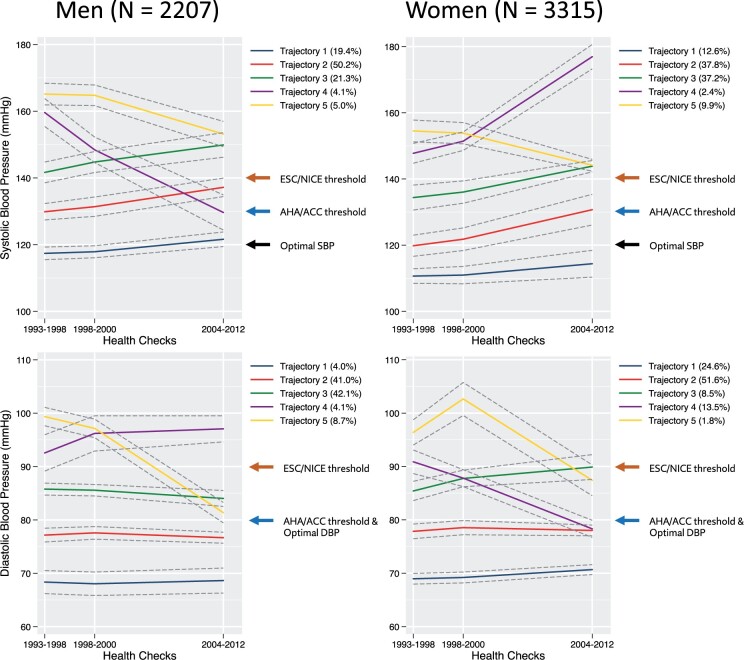
Long-term blood pressure patterns amongst men (*n* = 2207) and women (*n* = 3315) participants included in the incident cardiovascular disease analyses. SBP trajectories in men: Trajectory 1—borderline stable optimal SBP, Trajectory 2—rising normal/high normal SBP, Trajectory 3—rising grade 1 hypertension, Trajectory 4—grade 1 hypertension → high-normal SBP, Trajectory 5—grade 2 → grade 1 hypertension. SBP trajectories in women: Trajectory 1—stable optimal SBP, Trajectory 2—rising normal/high normal SBP, Trajectory 3—high-normal SBP → grade 1 hypertension, Trajectory 4—decreasing grade 1 hypertension, Trajectory 5—grade 1 → grade 2 hypertension. DBP trajectories: Trajectory 1—low optimal DBP, Trajectory 2—high optimal DBP, Trajectory 3—normal/high-normal DBP, Trajectory 4—grade 1 hypertension → normal DBP, Trajectory 5—stable grade 1/grade 2 hypertension. 95% confidence intervals are represented as dashed grey lines.

Amongst men included in the incident CVD analyses, 5 SBP trajectories were revealed (*Figure 3* and Supplementary material online, *Table S3*): 19.4% belonged to trajectory 1 (borderline stable optimal SBP), 50.2% to trajectory 2 (rising normal/high normal SBP), 21.3% to trajectory 3 (rising grade 1 hypertension), 4.1% to trajectory 4 (grade 1 hypertension → high-normal SBP), and 5.0% to trajectory 5 (grade 2 → grade 1 hypertension). Amongst women included in the incident CVD analyses, there were five similar SBP trajectories: 12.6% belonged to trajectory 1 (stable optimal SBP), 37.8% to trajectory 2 (rising normal/high-normal SBP), 37.2% to trajectory 3 (high-normal SBP → grade 1 hypertension), 2.4% to trajectory 4 (grade 1 → grade 2 hypertension), and 9.9% to trajectory 5 (decreasing grade 1 hypertension) and

All analysed groups revealed five distinct DBP trajectories, which were similar in all groups: trajectory 1 (low optimal DBP), trajectory 2 (high optimal DBP), trajectory 3 (normal/high-normal DBP), trajectory 4 (grade 1 hypertension → normal DBP), and trajectory 5 (stable grade 1/grade 2 hypertension).

### Cox regression analyses

**Figure 4:**
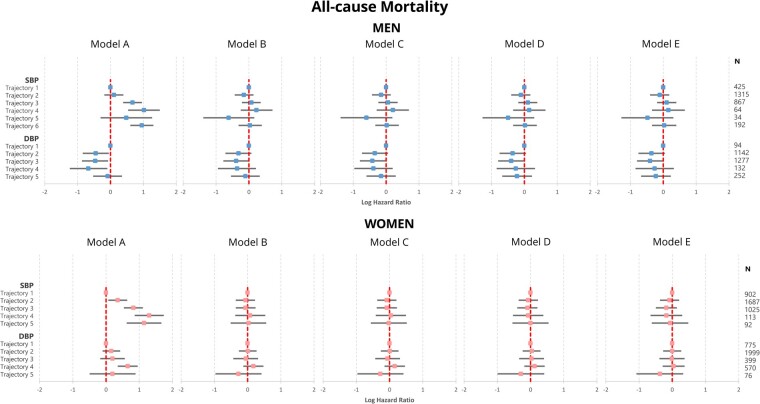
Results of Cox regressions assessing the association between blood pressure phenotypes and all-cause mortality. Model A—Univariable; Model B—Multivariable adjustment for age and ethnicity; Model C—Model B + body mass index, physical activity level, smoking and alcohol consumption; Model D—Model C + pre-existing co-morbidities (cardiovascular disease, diabetes mellitus, cancer, asthma, chronic obstructive pulmonary disease) and serum low-density lipoprotein cholesterol; Model E—Model D + antihypertensive treatment. SBP trajectories in men: Trajectory 1—borderline stable optimal SBP, Trajectory 2—rising normal/high normal SBP, Trajectory 3—rising grade 1 hypertension, Trajectory 4—grade 1 hypertension → high-normal SBP, Trajectory 5—grade 2 → grade 1 hypertension. SBP trajectories in women: Trajectory 1—stable optimal SBP, Trajectory 2—rising normal/high normal SBP, Trajectory 3—high-normal SBP → grade 1 hypertension, Trajectory 4—decreasing grade 1 hypertension, Trajectory 5—grade 1 → grade 2 hypertension. DBP trajectories: Trajectory 1—low optimal DBP, Trajectory 2—high optimal DBP, Trajectory 3—normal/high-normal DBP, Trajectory 4—grade 1 hypertension → normal DBP, Trajectory 5—stable grade 1/grade 2 hypertension. Median (interquartile range) follow-up was 9.4 (8.0–10.9) years amongst both men and women, respectively. DBP, diastolic blood pressure; SBP, systolic blood pressure.

Median follow-up periods (interquartile range) were 9.4 (8.0–10.9) and 9.4 (7.8–11.0) years for men and women, respectively. Univariable analyses (Model A) showed that compared to SBP trajectory 1 participants (stable optimal SBP), the other SBP trajectories were associated with increased risk of incident all-cause, cardiovascular, and non-cardiovascular mortality amongst both sexes (*Figure 4* and Supplementary material online, *Tables S4*–*S6*). In men, trajectories 3–6 were associated with two-fold increases in the risk of all-cause mortality, while trajectories 2–6 were associated with up to three-fold increases for the same outcome (*Figure 4*). Nevertheless, these associations with any of the mortality outcomes were not revealed upon confounder adjustment (Models B–E). In men, similar patterns were revealed between DBP trajectories and incident mortality. In univariable analyses (Model A), trajectories 2–4 were associated with 35–50% reductions in the risk of incident all-cause mortality compared to trajectory 1 (low optimal DBP), which were also not revealed upon confounder adjustment (Models B–E). Nevertheless, there were a few significant associations between DBP trajectories and cardiovascular mortality amongst men. Compared to trajectory 1 (low optimal DBP), trajectories 2 (high optimal DBP), and 3 (normal/high-normal DBP) were associated with significantly lower cardiovascular mortality in all models considered (Models A–E).

The sensitivity all-cause mortality analysis considering only participants without prevalent CVD at the third health checked revealed results consistent with the main analyses (Supplementary material online, *Table S7*).

**Figure 5:**
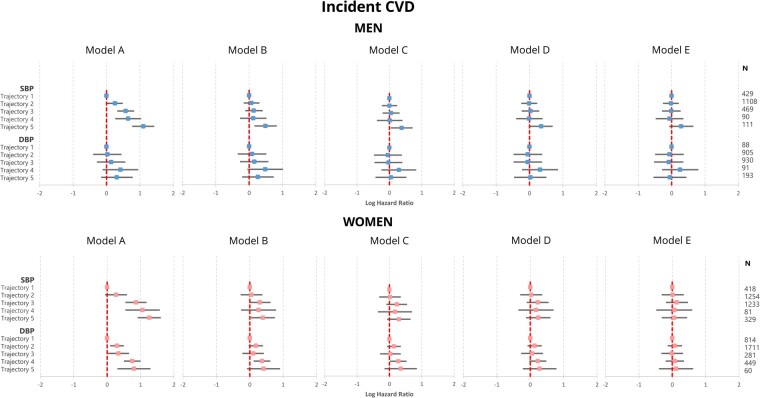
Results of Cox regressions assessing the association between blood pressure phenotypes and incident cardiovascular disease amongst participants without prevalent cardiovascular disease at the third health check of the EPIC-Norfolk study. Model A—Univariable; Model B—Multivariable adjustment for age and ethnicity; Model C—Model B + body mass index, physical activity level, smoking and alcohol consumption; Model D—Model C + pre-existing co-morbidities (cardiovascular disease, diabetes mellitus, cancer, asthma, chronic obstructive pulmonary disease) and serum low-density lipoprotein cholesterol; Model E—Model D + antihypertensive treatment. SBP trajectories in men: Trajectory 1—borderline stable optimal SBP, Trajectory 2—rising normal/high normal SBP, Trajectory 3—rising grade 1 hypertension, Trajectory 4—grade 1 hypertension → high-normal SBP, Trajectory 5—grade 2 → grade 1 hypertension. SBP trajectories in women: Trajectory 1—stable optimal SBP, Trajectory 2—rising normal/high normal SBP, Trajectory 3—high-normal SBP → grade 1 hypertension, Trajectory 4—decreasing grade 1 hypertension, Trajectory 5—grade 1 → grade 2 hypertension. DBP trajectories: Trajectory 1—low optimal DBP, Trajectory 2—high optimal DBP, Trajectory 3—normal/high-normal DBP, Trajectory 4—grade 1 hypertension → normal DBP, Trajectory 5—stable grade 1/grade 2 hypertension. Median (interquartile range) follow-up was 9.4 (8.0–10.9) and 9.4 (7.8–11.0) years amongst men and women, respectively. DBP, diastolic blood pressure; SBP, systolic blood pressure.

Amongst men, compared to SBP trajectory 1 (stable optimal SBP), trajectories 2–5 were associated with significant increases between 30% and three-fold in incident CVD in the univariable model (Model A). The associations between trajectories 2–4 and incident CVD were not revealed after confounder adjustment (Models B–E). Nevertheless, the association between trajectory 5 (grade 2 → grade 1 hypertension) was revealed upon adjustment for age, ethnicity, BMI, physical activity level, smoking, and alcohol consumption and pre-existing comorbidities (Models B–D), but not after further adjustment for antihypertensive treatment (Model E) (*Figure 5*, Supplementary material online, *Table S8*). There were no statistically significant relationships between DBP trajectories 2-5 (compared to trajectory 1) and incident CVD amongst men across all fully adjusted models considered.

Amongst women, compared to SBP trajectory 1 (stable optimal SBP), trajectories 3–5 were associated with significant two- to three-fold increases in the risk of incident CVD at univariable level (Model A). The associations between trajectories 2–4 and incident CVD were not revealed upon confounder adjustment (Models B–E). Nevertheless, the association between trajectory 5 (decreasing grade 1 hypertension) and incident CVD was revealed upon adjustment for age and ethnicity (Model B), but not after further confounder adjustment (Models C–E). Compared to DBP trajectory 1 (low optimal DBP), trajectories 2–5 were associated with significant 30% to two-fold increases in the risk of incident CVD amongst women. The associations between trajectories 2, 3, and 5 and incident CVD were not revealed upon confounder adjustment (Models B–E), while the association between trajectory 4 (grade 1 hypertension → normal DBP) and incident CVD was revealed upon adjustment for age, ethnicity, BMI, physical activity and smoking, alcohol consumption (Models B–C) but not after further adjustment for pre-existing comorbidities and antihypertensive treatment (Models D–E).

## Discussion

In this prospective cohort study with long-term historical BP data spanning ∼13 years, we determined the sex differences in longitudinal BP trajectories in mid-life and have delineated their associations with mortality (all-cause, cardiovascular, and non-cardiovascular) and incident CVD in later life. We found that women were significantly younger, but demonstrated a worse cardiometabolic profile at baseline with higher baseline LDL-c levels, lower levels of physical activity, and higher prevalence of smoking. Women were also less likely to receive primary CVD prevention, antihypertensive, and anti-diabetic treatment. They nevertheless tended to have SBP trajectories characterized by lower mean measurements, while men tended to exhibit more pronounced decreases in SBP over the duration of the exposure period. Despite overall lower mortality and incident CVD rates amongst women, the excess risk of these adverse outcomes associated with hypertensive trajectories was higher in women than in men at univariable level. While the univariable associations between most longitudinal BP trajectories and mortality were rendered non-significant upon only age and ethnicity adjustment amongst both sexes, the association between hypertensive BP trajectories and incident mortality amongst men was maintained after comprehensive adjustment for age, ethnicity, lifestyle factors, comorbidities, and baseline LDL-c levels. Further adjustment for anti-hypertensive treatment rendered this association non-significant, suggesting that the long-term adverse effects of hypertension may be offset by appropriate and timely antihypertensive treatment.

Several previous investigations assessed the relationship between long-term BP burden and CVD. A dose–response relationship between cumulative exposure to elevated BP and incident CVD and cardiovascular mortality has been previously reported.^13^^,^^28–31^ Furthermore, several investigations have reported that prolonged exposure to elevated BP trajectories is associated with increased atherosclerotic burden,^22^ incident stroke,^7^ heart failure,^9^ AF,^11^ overall CVD,^9^ all-cause mortality,^24^ and cardiovascular mortality.^12^ Recent findings indicate that long-term BP trajectories may differ by sex.^15^ Indeed, sex differences have also been recently reported for incident AF.^11^ Amongst 7670 men and 8376 women from a Norwegian prospective cohort with a mean age at the beginning of the exposure period of ∼40 years (mean age of our cohort at the beginning of the exposure period was 56.2 years), stronger associations between elevated/hypertensive BP trajectories and incident AF were documented amongst women than in men.^11^ However, no previous investigations have assessed the relationship between the different phenotypes of longitudinal BP changes and adverse outcomes separately amongst men and women. Our study is the first to report these relationships.

Our results suggest that men exhibit higher SBP trajectories than women, with men in the reference SBP trajectory having a mean SBP of ∼120 mmHg throughout the exposure period while women in the reference group ∼110 mmHg. Nevertheless, more pronounced SBP decreases were recorded in men. This may be attributed to a tendency of undertreating hypertension in women, illustrated by lower utilization of antihypertensive agents in women than in men at the end of the exposure period (36% vs. 42%, respectively). Our results are largely consistent with previous findings suggesting a larger unused potential for cardiovascular prevention by BP reduction strategies in women than in men.^32^ Furthermore, we also found that compared to low optimal DBP (<70 mmHg), DBP trajectories characterized by high normal values (80–90 mmHg) were associated with lower risk of all-cause and cardiovascular mortality in men, even after full multivariable adjustment. Such associations were nevertheless not revealed in women (who had lower CVD prevalence at baseline) or in participants included in the incident CVD analyses (who were free of prevalent CVD at baseline), suggesting that long-term exposure DBP <70 mmHg may be deleterious in patients with pre-existing CVD, which has been previously reported in larger studies with shorter follow-up.^33^^,^^34^

The overall lack of association between the other BP trajectories and outcomes may be related to the characteristics of the included participant sample: mean BMI ∼26.5–26.9 kg/m^2^, estimated glomerular filtration rate ∼73 mL/min/1.73 m^2^, glycated haemoglobin levels ∼5.8% (39.9 mmol/mol), LDL cholesterol levels ∼3.0 mmol/L, ∼95% non-smokers. Furthermore, a high proportion of participants were undergoing CVD primary prevention with aspirin, lipid-lowering, and antihypertensive agents at the end of the exposure period. The included sample thus comprised of relatively healthy participants undergoing appropriate primary prevention in whom incident CVD would be less likely to result in a fatal event. This may also be explained by survivorship bias, in which the inclusion of a healthier sub-population surviving over a ∼13-year-old period spanning the first three HCs of the EPIC-Norfolk study may have led to the underestimation of the mortality and incident CVD risk. Further studies replicating our analytic strategy are therefore warranted to determine the same associations in other cohorts with differing distributions of ethnicity, cardiovascular risk profile and comorbidities to ensure the external validity of our findings.

Our results may inform primary CVD prevention practice by highlighting the importance of sex differences in the natural course and management of hypertension from mid-life onwards. While women exhibited BP trajectories characterized by initial lower values, men received more aggressive antihypertensive therapy, resulting in more pronounced longitudinal BP decreases. Suboptimal primary CVD prevention amongst women is also reflected in poorer cardiometabolic health at baseline and higher relative risk increases associated with exposure to non-optimal BP values in women at univariable level. These results therefore reflect important between-sex disparities in primary prevention which need to be addressed in order to ensure appropriate and fair provision of care. Therefore, a programme of systematic population screening for hypertension with a special emphasis on early diagnosis in women should be implemented in order to address the sex disparities highlighted by our study. Patient education and regular follow-up are also be warranted to ensure appropriate compliance with antihypertensive therapy. Furthermore, the results of our study in addition to previous literature^33^^,^^34^ highlight that lowering DBP to values <70 mmHg may be deleterious in men as well as patients with pre-existing CVD and such stringent BP treatment targets should be avoided in these populations.

Our study benefited from several strengths. We used data from EPIC-Norfolk, a large, prospective cohort with robust ascertainment of exposures, confounders and outcomes. Furthermore, EPIC-Norfolk benefits from long-term follow-up, allowing the determination of long-term BP trajectories over 12 years and the adjudication of outcomes over a median follow-up of 9.4 years after the exposure period. We were able to control for wide range of important demographic, lifestyle, social and medical factors. Furthermore, we employed robust statistical methods to adjudicate longitudinal BP trajectory group membership.

Naturally, there were also limitations. We included >99% White Caucasian participants, and thus ethnicity-stratified analyses could not be performed. Further studies are required to determine these associations amongst other ethnicities. BP trajectories were adjudicated based on measurements from three HCs. Nevertheless, no other BP measurements were acquired and therefore BP variations occurring between these HCs were not considered, introducing potential uncertainty regarding the development of BP trajectories between the HCs. Furthermore, we used self-reported comorbidities, which may lead to inaccuracies. We nevertheless employed a combined comorbidity definition also employing medication data, biomarkers measured at the HCs and incident diagnoses reported during the exposure period in order to minimize potential inaccuracies in the ascertainment of self-reported comorbidities. Furthermore, having only included a subgroup of the EPIC-Norfolk prospective study which attended the first three HCs of the study spanning ∼13 years, our analyses may be prone to survivorship bias. Nevertheless, this is an inherent limitation of any study analysing longitudinal changes in BP. Despite adjusting for a wide range of participant characteristics at the beginning of the follow-up, data describing the evolution of these co-variates over the follow-up were not available, which did not allow longitudinal co-variate changes to be considered. As an observational study, we were not able to account for residual confounders.

In conclusion, using data from a large-scale prospective cohort study, we determined the sex differences in longitudinal BP trajectories in midlife and delineated the associations between these and mortality and incident CVD in older age. While men exhibited higher BP longitudinal trajectories than women, these were characterized by more pronounced BP decreases throughout the exposure period of the study, which may be attributed to the relative undertreatment of women. This suggests important between-sex disparities in primary CVD prevention. Exposure to hypertensive SBP and DBP trajectories was associated with higher mortality and incident CVD risk amongst both sexes at univariable level. Nevertheless, these associations were not maintained upon extensive confounder adjustment including antihypertensive therapy, suggesting that effective therapy may offset the adverse outcomes associated with prolonged exposure to high BP. Our results also highlight that longitudinal exposure to low DBP values <70 mmHg may be independently associated with higher risk of all-cause and cardiovascular mortality amongst men.

## Supplementary material

Supplementary material is available at *European Journal of Preventive Cardiology* online.

## Supplementary Material

zwab104_Supplementary_Data
