# Supplementary material for: Long Term Prognostic Impact of Sex-specific Longitudinal Changes in Blood Pressure. The EPIC-Norfolk Prospective Population Cohort Study
Source: Eur J Prev Cardiol. 2021 Jul 5;29(1):180–91. doi: 10.1093/eurjpc/zwab104 (PMC8858020; doi:10.1093/eurjpc/zwab104)
Supplement: zwab104_Supplementary_Data [file zwab104_supplementary_data.docx]

**SUPPLEMENTARY MATERIAL**

**Supplementary Tables**

**Supplementary Table 1**. Third health check characteristics of included participants from the European Prospective Investigation in Cancer (EPIC)-Norfolk (unless otherwise stated), stratified by whether data on third health check physical activity levels, smoking status, units of alcohol drunk, serum creatinine, glycated haemoglobin or low-density lipoprotein cholesterol were missing.

|  | **Not missing** | **Missing** | **P value** |
| --- | --- | --- | --- |
|  | 4352 | 2364 |  |
| Age, mean (SD) | 69.81 (7.70) | 67.96 (8.86) | **<0.001** |
| Sex, N(%) |  |  | 0.450 |
| Men | 1892 (43.47) | 1005 (42.51) |  |
| Women | 2460 (56.53) | 1359 (57.49) |  |
| Ethnicity, N(%) |  |  | 0.170 |
| White | 4336 (99.63) | 2357 (99.70) |  |
| Black, Caribbean | 1 (0.02) | 2 (0.09) |  |
| Black, other | 1 (0.02) | 0 (0.00) |  |
| Indian | 3 (0.07) | 0 (0.00) |  |
| Pakistani | 0 (0.00) | 2 (0.09) |  |
| Chinese | 3 (0.07) | 0 (0.00) |  |
| Other | 8 (0.18) | 3 (0.13) |  |
| Weight (kg), mean (SD) | 73.46 (13.63) | 74.24 (14.33) | 0.029 |
| Height (cm), mean (SD) | 166.14 (9.01) | 166.12 (9.07) | 0.942 |
| Body Mass Index (kg/m^2^), mean (SD) | 26.54 (4.08) | 26.83 (4.34) | 0.008 |
| Systolic Blood Pressure  (mmHg), mean (SD) | 136.18 (16.55) | 136.33 (16.17) | 0.728 |
| Diastolic Blood Pressure  (mmHg), mean (SD) | 77.93 (9.33) | 78.49 (9.27) | 0.019 |
| Heart Rate (beats/min), mean (SD) | 68.75 (14.17) | 69.73 (11.20) | 0.004 |
| Educational Level, N (%) |  |  | **<0.001** |
| None | 1051 (24.15) | 699 (29.57) |  |
| O-level | 512 (11.76) | 289 (12.23) |  |
| A-level | 1944 (44.67) | 1021 (43.19) |  |
| University Degree | 845 (19.42) | 355 (15.02) |  |
| **Pre-existing co-morbidities** |  |  |  |
| Cerebrovascular disease, N (%) | 54 (1.24) | 33 (1.4) | 0.591 |
| Diabetes mellitus, N (%) | 109 (2.5) | 72 (3.05) | 0.191 |
| Myocardial infarction, N(%) | 114 (2.62) | 63 (2.66) | 0.912 |
| Cancer, N(%) | 343 (7.88) | 204 (8.63) | 0.284 |
| Asthma, N (%) | 430 (9.88) | 207 (8.76) | 0.133 |
| Chronic Obstructive Pulmonary Disease, N (%) | 384 (8.82) | 194 (8.21) | 0.389 |
| **Drug Therapy** |  |  |  |
| Aspirin, N (%) | 832 (19.12) | 426 (18.02) | 0.271 |
| Lipid-lowering agents, N (%) | 1082 (24.86) | 462 (19.54) | **<0.001** |
| Non-steroidal anti-inflammatory drugs, N (%) | 1125 (25.85) | 577 (24.41) | 0.194 |
| Anti-diabetic drugs, N (%) | 166 (3.81) | 105 (4.44) | 0.212 |
| Antihypertensive agents, N (%) | 1680 (38.6) | 913 (38.62) | 0.988 |
| ACE Inhibitors, N (%) | 754 (17.33) | 348 (14.72) | **0.006** |
| Beta-Blockers, N (%) | 530 (12.18) | 318 (13.45) | 0.133 |
| Loop Diuretics, N (%) | 183 (4.2) | 106 (4.48) | 0.591 |
| Other Diuretics, N (%) | 554 (12.73) | 335 (14.17) | 0.096 |
| Angiotensin Receptor Blockers, N (%) | 313 (7.19) | 125 (5.29) | **0.003** |
| Calcium Channel Blockers, N (%) | 562 (12.91) | 273 (11.55) | 0.105 |
| **Incident Outcomes†** | | | |
| Mortality, N (%) |  |  |  |
| All-cause | 593 (13.63) | 455 (19.25) | **<0.001** |
| Cardiovascular | 168 (3.86) | 125 (5.29) | **0.006** |
| Non-cardiovascular | 425 (9.77) | 330 (13.96) | **<0.001** |
| Incident cardiovascular disease‡, N(%) | 1151 (35.51) | 734 (41.31) | **<0.001** |

SD – standard deviation, IQR – interquartile range, ACE - Angiotensin-converting enzyme;

**†**Incident outcomes measured during the follow-up period from the third health check (2004-2012) until the end of March 2018, resulting in a median follow-up of 9.44 years.

‡Incident cardiovascular disorders reported only amongst patients without pre-existing cardiovascular disease at the third health check (N = 5018).

Statistically significant results (*P* < 0.05) are highlighted in **bold**.

**Supplementary Table 2.** Blood pressure trajectories amongst men (n = 2897) and women (n = 3819) participants included in the mortality analyses.

|  | Health Check 1  (1993-1998) | Health Check 2  (1998-2000) | Health Check 3  (2004-2012) |
| --- | --- | --- | --- |
| **MEN** | | | |
| **Systolic Blood Pressure (95% Confidence Interval), mmHg** | | | |
| Trajectory 1 | 117.17 (115.29 - 119.06) | 117.10 (115.13 - 119.07) | 119.25 (117.05 - 121.45) |
| Trajectory 2 | 128.70 (119.06 - 126.68) | 129.79 (119.07 - 127.60) | 134.91 (121.45 - 132.42) |
| Trajectory 3 | 141.72 (126.68 - 130.72) | 143.81 (127.60 - 131.98) | 145.75 (132.42 - 137.40) |
| Trajectory 4 | 156.85 (130.72 - 139.35) | 145.33 (131.98 - 141.53) | 118.40 (137.40 - 144.19) |
| Trajectory 5 | 148.28 (139.35 - 144.08) | 152.45 (141.53 - 146.10) | 180.53 (144.19 - 147.32) |
| Trajectory 6 | 165.66 (144.08 - 152.01) | 163.51 (146.10 - 140.56) | 145.70 (147.32 - 112.01) |
| **Diastolic Blood Pressure (95% Confidence Interval), mmHg** | | | |
| Trajectory 1 | 68.44 (66.21 - 70.68) | 67.46 (65.20 - 69.72) | 67.00 (64.46 - 69.55) |
| Trajectory 2 | 77.29 (70.68 - 75.89) | 77.19 (69.72 - 75.87) | 75.68 (69.55 - 74.55) |
| Trajectory 3 | 85.81 (75.89 - 78.69) | 85.59 (75.87 - 78.52) | 82.19 (74.55 - 76.81) |
| Trajectory 4 | 91.66 (78.69 - 84.58) | 94.35 (78.52 - 84.34) | 95.49 (76.81 - 80.23) |
| Trajectory 5 | 100.33 (84.58 - 87.05) | 97.57 (84.34 - 86.84) | 79.76 (80.23 - 84.15) |
| **WOMEN** | | | |
| **Systolic Blood Pressure (95% Confidence Interval), mmHg** | | | |
| Trajectory 1 | 113.01 (111.94 - 114.08) | 113.82 (112.72 - 114.92) | 118.48 (116.96 - 120.00) |
| Trajectory 2 | 125.32 (114.08 - 123.62) | 127.26 (114.92 - 125.59) | 137.17 (120.00 - 135.45) |
| Trajectory 3 | 142.96 (123.62 - 127.02) | 142.95 (125.59 - 128.93) | 143.77 (135.45 - 138.89) |
| Trajectory 4 | 163.67 (127.02 - 140.52) | 163.11 (128.93 - 140.91) | 144.27 (138.89 - 142.45) |
| Trajectory 5 | 146.09 (140.52 - 145.40) | 153.34 (140.91 - 145.00) | 179.20 (142.45 - 145.09) |
| **Diastolic Blood Pressure (95% Confidence Interval), mmHg** | | | |
| Trajectory 1 | 68.64 (67.49 - 69.78) | 68.90 (67.77 - 70.03) | 70.23 (69.23 - 71.23) |
| Trajectory 2 | 77.49 (69.78 - 76.13) | 77.71 (70.03 - 76.40) | 77.11 (71.23 - 76.07) |
| Trajectory 3 | 84.77 (76.13 - 78.84) | 87.12 (76.40 - 79.01) | 88.56 (76.07 - 78.15) |
| Trajectory 4 | 90.88 (78.84 - 83.19) | 87.46 (79.01 - 85.59) | 77.36 (78.15 - 86.18) |
| Trajectory 5 | 96.64 (83.19 - 86.36) | 102.73 (85.59 - 88.65) | 87.77 (86.18 - 90.94) |

**Supplementary Table 3.** Blood pressure trajectories amongst men (n = 2207) and women (n = 3315) participants without prevalent cardiovascular disease at the third health check included in the incident cardiovascular disease analyses.

|  | Health Check 1  (1993-1998) | Health Check 2  (1998-2000) | Health Check 3  (2004-2012) |
| --- | --- | --- | --- |
| **MEN** | | | |
| **Systolic Blood Pressure (95% Confidence Interval), mmHg** | | | |
| Trajectory 1 | 117.39 (115.49 - 119.29) | 117.88 (116.08 - 119.68) | 121.62 (119.43 - 123.80) |
| Trajectory 2 | 129.87 (119.29 - 127.41) | 131.39 (119.68 - 128.49) | 137.18 (123.80 - 134.41) |
| Trajectory 3 | 141.67 (127.41 - 132.33) | 144.78 (128.49 - 134.29) | 149.89 (134.41 - 139.95) |
| Trajectory 4 | 159.60 (132.33 - 138.56) | 148.42 (134.29 - 141.64) | 129.65 (139.95 - 146.21) |
| Trajectory 5 | 165.15 (138.56 - 144.77) | 164.77 (141.64 - 147.93) | 153.16 (146.21 - 153.57) |
| **Diastolic Blood Pressure (95% Confidence Interval), mmHg** | | | |
| Trajectory 1 | 68.36 (66.19 - 70.52) | 68.04 (65.84 - 70.24) | 68.64 (66.30 - 70.98) |
| Trajectory 2 | 77.16 (70.52 - 75.87) | 77.57 (70.24 - 76.39) | 76.67 (70.98 - 75.64) |
| Trajectory 3 | 85.78 (75.87 - 78.45) | 85.57 (76.39 - 78.75) | 84.02 (75.64 - 77.70) |
| Trajectory 4 | 92.56 (78.45 - 84.66) | 96.21 (78.75 - 84.49) | 97.07 (77.70 - 82.53) |
| Trajectory 5 | 99.36 (84.66 - 86.90) | 97.14 (84.49 - 86.64) | 81.37 (82.53 - 85.52) |
| **WOMEN** | | | |
| **Systolic Blood Pressure (95% Confidence Interval), mmHg** | | | |
| Trajectory 1 | 110.67 (108.46 - 112.88) | 110.97 (108.34 - 113.60) | 114.40 (110.34 - 118.45) |
| Trajectory 2 | 119.83 (112.88 - 116.64) | 121.81 (113.60 - 118.39) | 130.73 (118.45 - 126.09) |
| Trajectory 3 | 134.38 (116.64 - 123.02) | 136.05 (118.39 - 125.22) | 143.87 (126.09 - 135.36) |
| Trajectory 4 | 147.76 (123.02 - 130.61) | 151.41 (125.22 - 132.68) | 176.94 (135.36 - 142.16) |
| Trajectory 5 | 154.52 (130.61 - 138.16) | 153.83 (132.68 - 139.42) | 144.14 (142.16 - 145.57) |
| **Diastolic Blood Pressure (95% Confidence Interval), mmHg** | | | |
| Trajectory 1 | 68.96 (67.96 - 69.96) | 69.20 (68.19 - 70.22) | 70.68 (69.75 - 71.61) |
| Trajectory 2 | 77.85 (69.96 - 76.48) | 78.54 (70.22 - 77.21) | 78.02 (71.61 - 77.01) |
| Trajectory 3 | 85.41 (76.48 - 79.21) | 87.76 (77.21 - 79.87) | 89.89 (77.01 - 79.02) |
| Trajectory 4 | 90.87 (79.21 - 83.61) | 87.81 (79.87 - 86.21) | 78.31 (79.02 - 87.56) |
| Trajectory 5 | 96.38 (83.61 - 87.22) | 102.65 (86.21 - 89.31) | 87.41 (87.56 - 92.22) |

**Supplementary Table 4.** Results of Cox proportional hazards regression models assessing the association between blood pressure phenotypes and all-cause mortality.

| **All-cause Mortality** | | | | | |
| --- | --- | --- | --- | --- | --- |
| **MEN** | | | | | |
| **Systolic Blood Pressure Trajectories** | | | | | |
| Hazard Ratio (95% Confidence Interval) | | | | | |
|  | Model A (Univariable) | Model B | Model C | Model D | Model E |
| Trajectory 1 | Reference | Reference | Reference | Reference | Reference |
| Trajectory 2 | 1.11 (0.83-1.48) | 0.86 (0.65-1.15) | 0.86 (0.65-1.15) | 0.90 (0.67-1.20) | 0.90 (0.67-1.20) |
| Trajectory 3 | **1.96 (1.47-2.60)** | 1.08 (0.81-1.43) | 1.06 (0.79-1.42) | 1.11 (0.83-1.48) | 1.11 (0.83-1.49) |
| Trajectory 4 | **2.77 (1.71-4.48)** | 1.26 (0.78-2.05) | 1.23 (0.75-2.00) | 1.16 (0.71-1.90) | 1.17 (0.71-1.93) |
| Trajectory 5 | 1.62 (0.74-3.55) | 0.54 (0.25-1.18) | 0.55 (0.25-1.21) | 0.61 (0.28-1.35) | 0.62 (0.28-1.36) |
| Trajectory 6 | **2.60 (1.83-3.69)** | 1.03 (0.73-1.48) | 1.02 (0.72-1.47) | 1.02 (0.71-1.46) | 1.03 (0.71-1.49) |
| **Diastolic Blood Pressure Trajectories** | | | | | |
| Trajectory 1 | Reference | Reference | Reference | Reference | Reference |
| Trajectory 2 | **0.64 (0.43-0.95)** | 0.73 (0.49-1.08) | 0.71 (0.48-1.06) | 0.70 (0.47-1.05) | 0.70 (0.47-1.05) |
| Trajectory 3 | **0.63 (0.42-0.93)** | 0.68 (0.46-1.01) | **0.66 (0.45-0.99)** | **0.67 (0.45-1.00)** | **0.67 (0.45-0.99)** |
| Trajectory 4 | **0.51 (0.29-0.91)** | 0.70 (0.39-1.24) | 0.68 (0.38-1.22) | 0.77 (0.43-1.38) | 0.77 (0.43-1.38) |
| Trajectory 5 | 0.92 (0.59-1.42) | 0.90 (0.58-1.39) | 0.86 (0.55-1.34) | 0.80 (0.51-1.26) | 0.80 (0.51-1.26) |
| **WOMEN** | | | | | |
| **Systolic Blood Pressure Trajectories** | | | | | |
|  | Model A (Univariable) | Model B | Model C | Model D | Model E |
| Trajectory 1 | Reference | Reference | Reference | Reference | Reference |
| Trajectory 2 | **1.42 (1.07-1.89)** | 0.94 (0.70-1.25) | 0.92 (0.69-1.23) | 0.93 (0.70-1.25) | 0.92 (0.69-1.23) |
| Trajectory 3 | **2.28 (1.71-3.03)** | 0.94 (0.70-1.27) | 0.92 (0.68-1.25) | 0.91 (0.67-1.23) | 0.84 (0.61-1.15) |
| Trajectory 4 | **3.67 (2.37-5.68)** | 1.08 (0.69-1.70) | 1.04 (0.66-1.64) | 0.93 (0.59-1.47) | 0.84 (0.52-1.35) |
| Trajectory 5 | **3.14 (1.86-5.29)** | 1.03 (0.60-1.75) | 0.98 (0.57-1.68) | 1.00 (0.58-1.72) | 0.94 (0.54-1.62) |
| **Diastolic Blood Pressure Trajectories** | | | | | |
| Trajectory 1 | Reference | Reference | Reference | Reference | Reference |
| Trajectory 2 | 1.17 (0.90-1.53) | 1.01 (0.77-1.31) | 1.01 (0.77-1.31) | 1.04 (0.79-1.35) | 1.00 (0.76-1.31) |
| Trajectory 3 | 1.21 (0.84-1.76) | 0.95 (0.65-1.37) | 0.94 (0.65-1.37) | 1.03 (0.71-1.50) | 0.99 (0.68-1.45) |
| Trajectory 4 | **1.92 (1.42-2.59)** | 1.19 (0.88-1.61) | 1.17 (0.86-1.59) | 1.13 (0.83-1.53) | 1.04 (0.75-1.45) |
| Trajectory 5 | 1.21 (0.61-2.43) | 0.76 (0.38-1.52) | 0.76 (0.38-1.52) | 0.75 (0.37-1.51) | 0.69 (0.34-1.39) |

Model A – Univariable

Model B – Multivariable adjustment for age and ethnicity

Model C – Model B + body mass index, physical activity level, smoking and alcohol consumption

Model D – Model C + pre-existing co-morbidities (cardiovascular disease, diabetes mellitus, cancer, asthma, chronic obstructive pulmonary disease) and serum low-density lipoprotein cholesterol

Model E – Model D + antihypertensive treatment

Median (interquartile range) follow-up was 9.4 (8.0-10.9) years amongst both men and women.

Statistically significant results (*P* < 0.05) are highlighted in **bold**.

**Supplementary Table 5.** Results of Cox proportional hazards regression models assessing the association between blood pressure phenotypes and cardiovascular mortality.

| **Cardiovascular Mortality** | | | | | |
| --- | --- | --- | --- | --- | --- |
| **MEN** | | | | | |
| **Systolic Blood Pressure Trajectories** | | | | | |
| Hazard Ratio (95% Confidence Interval) | | | | | |
|  | Model A (Univariable) | Model B | Model C | Model D | Model E |
| Trajectory 1 | Reference | Reference | Reference | Reference | Reference |
| Trajectory 2 | 1.48 (0.77-2.83) | 1.12 (0.58-2.16) | 1.09 (0.57-2.10) | 1.16 (0.60-2.25) | 1.16 (0.60-2.24) |
| Trajectory 3 | **3.36 (1.78-6.33)** | 1.77 (0.93-3.37) | 1.68 (0.89-3.21) | **1.94 (1.02-3.71)** | **1.98 (1.03-3.80)** |
| Trajectory 4 | 2.65 (0.84-8.31) | 1.15 (0.36-3.63) | 1.04 (0.33-3.30) | 1.08 (0.34-3.44) | 1.12 (0.35-3.60) |
| Trajectory 5 | 2.55 (0.56-11.49) | 0.81 (0.18-3.66) | 0.80 (0.18-3.63) | 1.07 (0.23-4.87) | 1.10 (0.24-5.04) |
| Trajectory 6 | **4.31 (2.07-9.00)** | 1.63 (0.77-3.44) | 1.54 (0.72-3.25) | 1.60 (0.75-3.41) | 1.67 (0.78-3.60) |
| **Diastolic Blood Pressure Trajectories** | | | | | |
| Trajectory 1 | Reference | Reference | Reference | Reference | Reference |
| Trajectory 2 | **0.42 (0.22-0.83)** | **0.49 (0.25-0.96)** | **0.45 (0.23-0.89)** | **0.46 (0.23-0.92)** | **0.46 (0.23-0.92)** |
| Trajectory 3 | **0.45 (0.23-0.88)** | **0.49 (0.25-0.95)** | **0.44 (0.22-0.87)** | **0.47 (0.24-0.94)** | **0.48 (0.24-0.96)** |
| Trajectory 4 | 0.65 (0.26-1.59) | 0.92 (0.37-2.27) | 0.84 (0.34-2.09) | 1.06 (0.42-2.65) | 1.09 (0.43-2.75) |
| Trajectory 5 | 0.86 (0.41-1.80) | 0.84 (0.40-1.76) | 0.73 (0.34-1.56) | 0.70 (0.33-1.49) | 0.72 (0.33-1.56) |
| **WOMEN** | | | | | |
| **Systolic Blood Pressure Trajectories** | | | | | |
|  | Model A (Univariable) | Model B | Model C | Model D | Model E |
| Trajectory 1 | Reference | Reference | Reference | Reference | Reference |
| Trajectory 2 | **2.48 (1.25-4.91)** | 1.39 (0.70-2.76) | 1.28 (0.64-2.56) | 1.27 (0.64-2.54) | 1.22 (0.61-2.45) |
| Trajectory 3 | **5.35 (2.74-10.45)** | 1.62 (0.82-3.22) | 1.43 (0.71-2.86) | 1.42 (0.71-2.84) | 1.21 (0.60-2.46) |
| Trajectory 4 | **8.21 (3.42-19.73)** | 1.62 (0.66-3.96) | 1.39 (0.56-3.43) | 1.22 (0.49-3.06) | 0.99 (0.39-2.50) |
| Trajectory 5 | **6.85 (2.49-18.85)** | 1.50 (0.54-4.20) | 1.33 (0.47-3.74) | 1.34 (0.47-3.81) | 1.16 (0.41-3.31) |
| **Diastolic Blood Pressure Trajectories** | | | | | |
| Trajectory 1 | Reference | Reference | Reference | Reference | Reference |
| Trajectory 2 | 1.01 (0.61-1.68) | 0.82 (0.50-1.36) | 0.78 (0.47-1.29) | 0.81 (0.49-1.35) | 0.73 (0.44-1.23) |
| Trajectory 3 | 1.24 (0.62-2.48) | 0.87 (0.44-1.74) | 0.79 (0.39-1.59) | 0.91 (0.45-1.84) | 0.83 (0.41-1.69) |
| Trajectory 4 | **2.74 (1.62-4.64)** | 1.47 (0.87-2.50) | 1.32 (0.77-2.26) | 1.30 (0.76-2.23) | 1.06 (0.60-1.88) |
| Trajectory 5 | 1.43 (0.43-4.78) | 0.73 (0.22-2.47) | 0.65 (0.19-2.19) | 0.68 (0.20-2.29) | 0.55 (0.16-1.90) |

Model A – Univariable

Model B – Multivariable adjustment for age and ethnicity

Model C – Model B + body mass index, physical activity level, smoking and alcohol consumption

Model D – Model C + pre-existing co-morbidities (cardiovascular disease, diabetes mellitus, cancer, asthma, chronic obstructive pulmonary disease) and serum low-density lipoprotein cholesterol

Model E – Model D + antihypertensive treatment

Median (interquartile range) follow-up was 9.4 (8.0-10.9) years amongst both men and women.

Statistically significant results (*P* < 0.05) are highlighted in **bold**.

**Supplementary Table 6.** Results of Cox proportional hazards regression models assessing the association between blood pressure phenotypes and cardiovascular mortality.

| **Non-cardiovascular Mortality** | | | | | |
| --- | --- | --- | --- | --- | --- |
| **MEN** | | | | | |
| **Systolic Blood Pressure Trajectories** | | | | | |
| Hazard Ratio (95% Confidence Interval) | | | | | |
|  | Model A (Univariable) | Model B | Model C | Model D | Model E |
| Trajectory 1 | Reference | Reference | Reference | Reference | Reference |
| Trajectory 2 | 1.02 (0.74-1.41) | 0.81 (0.58-1.11) | 0.81 (0.59-1.12) | 0.84 (0.61-1.16) | 0.84 (0.61-1.16) |
| Trajectory 3 | **1.64 (1.19-2.26)** | 0.92 (0.66-1.27) | 0.92 (0.66-1.27) | 0.92 (0.66-1.28) | 0.92 (0.66-1.28) |
| Trajectory 4 | **2.79 (1.64-4.74)** | 1.29 (0.76-2.21) | 1.29 (0.75-2.21) | 1.18 (0.69-2.04) | 1.17 (0.67-2.04) |
| Trajectory 5 | 1.42 (0.56-3.56) | 0.48 (0.19-1.21) | 0.49 (0.20-1.25) | 0.51 (0.20-1.29) | 0.51 (0.20-1.29) |
| Trajectory 6 | **2.22 (1.48-3.32)** | 0.90 (0.60-1.35) | 0.91 (0.60-1.37) | 0.88 (0.58-1.33) | 0.87 (0.57-1.34) |
| **Diastolic Blood Pressure Trajectories** | | | | | |
| Trajectory 1 | Reference | Reference | Reference | Reference | Reference |
| Trajectory 2 | 0.76 (0.47-1.23) | 0.87 (0.53-1.41) | 0.86 (0.53-1.40) | 0.84 (0.52-1.37) | 0.84 (0.51-1.37) |
| Trajectory 3 | 0.73 (0.45-1.18) | 0.79 (0.49-1.29) | 0.79 (0.48-1.29) | 0.78 (0.47-1.27) | 0.77 (0.47-1.26) |
| Trajectory 4 | **0.44 (0.21-0.92)** | 0.59 (0.28-1.25) | 0.59 (0.28-1.26) | 0.64 (0.30-1.38) | 0.63 (0.30-1.36) |
| Trajectory 5 | 0.95 (0.55-1.64) | 0.93 (0.54-1.60) | 0.92 (0.53-1.60) | 0.85 (0.49-1.48) | 0.83 (0.47-1.46) |
| **WOMEN** | | | | | |
| **Systolic Blood Pressure Trajectories** | | | | | |
|  | Model A (Univariable) | Model B | Model C | Model D | Model E |
| Trajectory 1 | Reference | Reference | Reference | Reference | Reference |
| Trajectory 2 | 1.23 (0.90-1.69) | 0.86 (0.63-1.19) | 0.87 (0.63-1.20) | 0.89 (0.64-1.22) | 0.88 (0.63-1.21) |
| Trajectory 3 | **1.72 (1.24-2.38)** | 0.80 (0.57-1.13) | 0.82 (0.58-1.16) | 0.80 (0.57-1.13) | 0.77 (0.54-1.11) |
| Trajectory 4 | **2.84 (1.69-4.79)** | 0.99 (0.58-1.69) | 0.99 (0.57-1.71) | 0.89 (0.52-1.54) | 0.84 (0.48-1.49) |
| Trajectory 5 | **2.46 (1.32-4.60)** | 0.94 (0.50-1.78) | 0.93 (0.49-1.76) | 0.95 (0.50-1.80) | 0.91 (0.48-1.75) |
| **Diastolic Blood Pressure Trajectories** | | | | | |
| Trajectory 1 | Reference | Reference | Reference | Reference | Reference |
| Trajectory 2 | 1.24 (0.91-1.68) | 1.08 (0.79-1.48) | 1.11 (0.81-1.51) | 1.13 (0.83-1.55) | 1.12 (0.81-1.53) |
| Trajectory 3 | 1.20 (0.77-1.86) | 0.97 (0.63-1.51) | 1.00 (0.64-1.55) | 1.08 (0.69-1.69) | 1.06 (0.68-1.67) |
| Trajectory 4 | **1.59 (1.10-2.30)** | 1.05 (0.72-1.52) | 1.08 (0.74-1.57) | 1.02 (0.70-1.50) | 0.99 (0.66-1.48) |
| Trajectory 5 | 1.13 (0.49-2.63) | 0.76 (0.33-1.77) | 0.79 (0.34-1.85) | 0.76 (0.33-1.79) | 0.73 (0.31-1.74) |

Model A – Univariable

Model B – Multivariable adjustment for age and ethnicity

Model C – Model B + body mass index, physical activity level, smoking and alcohol consumption

Model D – Model C + pre-existing co-morbidities (cardiovascular disease, diabetes mellitus, cancer, asthma, chronic obstructive pulmonary disease) and serum low-density lipoprotein cholesterol

Model E – Model D + antihypertensive treatment

Median (interquartile range) follow-up was 9.4 (8.0-10.9) years amongst both men and women.

Statistically significant results (*P* < 0.05) are highlighted in **bold**.

**Supplementary Table 7.** Results of sensitivity Cox proportional hazards regression models assessing the association between blood pressure phenotypes and all-cause mortality amongst participants without prevalent cardiovascular disease at the third health check of the EPIC-Norfolk study.

| **All-cause Mortality (Sensitivity Analysis)** | | | | | |
| --- | --- | --- | --- | --- | --- |
| **MEN** | | | | | |
| **Systolic Blood Pressure Trajectories** | | | | | |
| Hazard Ratio (95% Confidence Interval) | | | | | |
|  | Model A (Univariable) | Model B | Model C | Model D | Model E |
| Trajectory 1 | Reference | Reference | Reference | Reference | Reference |
| Trajectory 2 | 1.29 (0.93-1.79) | 0.93 (0.67-1.29) | 0.93 (0.66-1.29) | 0.94 (0.67-1.31) | 0.94 (0.67-1.31) |
| Trajectory 3 | **1.90 (1.34-2.70)** | 0.92 (0.64-1.31) | 0.92 (0.64-1.32) | 0.88 (0.61-1.26) | 0.87 (0.60-1.27) |
| Trajectory 4 | **2.78 (1.72-4.49)** | 1.14 (0.70-1.85) | 1.12 (0.68-1.83) | 1.07 (0.65-1.76) | 1.06 (0.63-1.79) |
| Trajectory 5 | **2.27 (1.40-3.67)** | 0.80 (0.49-1.30) | 0.79 (0.49-1.30) | 0.79 (0.48-1.29) | 0.78 (0.46-1.32) |
| **Diastolic Blood Pressure Trajectories** | | | | | |
| Trajectory 1 | Reference | Reference | Reference | Reference | Reference |
| Trajectory 2 | 0.72 (0.43-1.19) | 0.83 (0.50-1.38) | 0.79 (0.47-1.32) | 0.79 (0.47-1.32) | 0.79 (0.48-1.33) |
| Trajectory 3 | 0.77 (0.46-1.27) | 0.85 (0.51-1.40) | 0.80 (0.47-1.33) | 0.79 (0.47-1.32) | 0.80 (0.47-1.36) |
| Trajectory 4 | 0.75 (0.36-1.55) | 0.91 (0.44-1.87) | 0.87 (0.42-1.81) | 0.90 (0.43-1.88) | 0.92 (0.44-1.95) |
| Trajectory 5 | 1.02 (0.58-1.81) | 1.02 (0.58-1.80) | 0.95 (0.53-1.70) | 0.91 (0.50-1.64) | 0.94 (0.51-1.74) |
| **WOMEN** | | | | | |
| **Systolic Blood Pressure Trajectories** | | | | | |
|  | Model A (Univariable) | Model B | Model C | Model D | Model E |
| Trajectory 1 | Reference | Reference | Reference | Reference | Reference |
| Trajectory 2 | **1.96 (1.19-3.24)** | 1.40 (0.85-2.31) | 1.42 (0.86-2.35) | 1.46 (0.88-2.42) | 1.44 (0.87-2.38) |
| Trajectory 3 | **2.54 (1.55-4.16)** | 1.16 (0.70-1.92) | 1.18 (0.71-1.95) | 1.19 (0.72-1.98) | 1.12 (0.67-1.88) |
| Trajectory 4 | **4.99 (2.51-9.90)** | 1.63 (0.81-3.27) | 1.64 (0.82-3.32) | 1.67 (0.82-3.38) | 1.55 (0.76-3.16) |
| Trajectory 5 | **4.34 (2.56-7.37)** | 1.33 (0.77-2.29) | 1.33 (0.77-2.31) | 1.27 (0.73-2.21) | 1.13 (0.63-2.00) |
| **Diastolic Blood Pressure Trajectories** | | | | | |
| Trajectory 1 | Reference | Reference | Reference | Reference | Reference |
| Trajectory 2 | 1.29 (0.97-1.72) | 1.11 (0.83-1.48) | 1.12 (0.84-1.50) | 1.12 (0.83-1.49) | 1.08 (0.81-1.45) |
| Trajectory 3 | 1.27 (0.81-1.99) | 0.94 (0.60-1.48) | 0.96 (0.61-1.51) | 0.98 (0.62-1.54) | 0.95 (0.60-1.50) |
| Trajectory 4 | **1.80 (1.27-2.56)** | 1.12 (0.79-1.59) | 1.13 (0.79-1.62) | 1.07 (0.74-1.53) | 0.99 (0.67-1.44) |
| Trajectory 5 | 1.46 (0.67-3.19) | 0.92 (0.42-2.02) | 0.91 (0.41-1.99) | 0.87 (0.39-1.91) | 0.79 (0.36-1.77) |

Model A – Univariable

Model B – Multivariable adjustment for age and ethnicity

Model C – Model B + body mass index, physical activity level, smoking and alcohol consumption

Model D – Model C + pre-existing co-morbidities (cardiovascular disease, diabetes mellitus, cancer, asthma, chronic obstructive pulmonary disease) and serum low-density lipoprotein cholesterol

Model E – Model D + antihypertensive treatment

Median (interquartile range) follow-up was 9.5 (8.1-11.0) and 9.5 (8.0-10.9) years amongst men and women, respectively.

Statistically significant results (*P* < 0.05) are highlighted in **bold**.

**Supplementary Table 8.** Results of univariable and multivariable Cox proportional hazards regression models assessing the association between blood pressure trajectories and incident cardiovascular disease amongst participants without prevalent cardiovascular disease at the third health check of the EPIC-Norfolk study.

| **Incident CVD** | | | | | |
| --- | --- | --- | --- | --- | --- |
| **MEN** | | | | | |
| **Systolic Blood Pressure Trajectories** | | | | | |
| Hazard Ratio (95% Confidence Interval) | | | | | |
|  | Model A (Univariable) | Model B | Model C | Model D | Model E |
| Trajectory 1 | Reference | Reference | Reference | Reference | Reference |
| Trajectory 2 | **1.29 (1.02-1.63)** | 1.07 (0.85-1.36) | 1.00 (0.79-1.27) | 0.99 (0.78-1.26) | 0.98 (0.77-1.24) |
| Trajectory 3 | **1.78 (1.38-2.29)** | 1.15 (0.89-1.50) | 1.05 (0.81-1.37) | 1.03 (0.79-1.34) | 0.99 (0.75-1.31) |
| Trajectory 4 | **1.91 (1.30-2.82)** | 1.13 (0.76-1.68) | 1.01 (0.68-1.50) | 0.99 (0.67-1.48) | 0.94 (0.62-1.42) |
| Trajectory 5 | **3.01 (2.16-4.18)** | **1.63 (1.17-2.29)** | **1.46 (1.04-2.05)** | **1.42 (1.01-2.00)** | 1.33 (0.93-1.92) |
| **Diastolic Blood Pressure Trajectories** | | | | | |
| Trajectory 1 | Reference | Reference | Reference | Reference | Reference |
| Trajectory 2 | 1.03 (0.67-1.57) | 1.09 (0.71-1.67) | 0.95 (0.62-1.47) | 0.95 (0.62-1.46) | 0.94 (0.61-1.44) |
| Trajectory 3 | 1.15 (0.75-1.76) | 1.17 (0.76-1.78) | 0.97 (0.63-1.49) | 0.95 (0.62-1.46) | 0.92 (0.59-1.42) |
| Trajectory 4 | 1.52 (0.89-2.59) | 1.62 (0.95-2.76) | 1.34 (0.78-2.29) | 1.37 (0.80-2.35) | 1.29 (0.75-2.23) |
| Trajectory 5 | 1.36 (0.85-2.19) | 1.30 (0.81-2.09) | 1.05 (0.65-1.69) | 1.03 (0.63-1.66) | 0.95 (0.58-1.56) |
| **WOMEN** | | | | | |
| **Systolic Blood Pressure Trajectories** | | | | | |
|  | Model A (Univariable) | Model B | Model C | Model D | Model E |
| Trajectory 1 | Reference | Reference | Reference | Reference | Reference |
| Trajectory 2 | 1.31 (0.94-1.81) | 1.05 (0.76-1.46) | 1.02 (0.73-1.42) | 1.03 (0.74-1.43) | 1.01 (0.73-1.41) |
| Trajectory 3 | **2.37 (1.73-3.25)** | 1.35 (0.98-1.86) | 1.26 (0.91-1.73) | 1.25 (0.90-1.73) | 1.15 (0.83-1.60) |
| Trajectory 4 | **2.88 (1.72-4.84)** | 1.30 (0.77-2.20) | 1.19 (0.70-2.01) | 1.19 (0.70-2.01) | 1.07 (0.62-1.82) |
| Trajectory 5 | **3.53 (2.49-4.99)** | **1.48 (1.03-2.12)** | 1.34 (0.93-1.92) | 1.27 (0.88-1.83) | 1.06 (0.73-1.56) |
| **Diastolic Blood Pressure Trajectories** | | | | | |
| Trajectory 1 | Reference | Reference | Reference | Reference | Reference |
| Trajectory 2 | **1.34 (1.09-1.65)** | 1.20 (0.97-1.48) | 1.15 (0.93-1.42) | 1.14 (0.92-1.41) | 1.08 (0.87-1.34) |
| Trajectory 3 | **1.40 (1.02-1.93)** | 1.11 (0.80-1.52) | 1.03 (0.75-1.42) | 1.06 (0.76-1.46) | 0.99 (0.72-1.38) |
| Trajectory 4 | **2.12 (1.65-2.72)** | **1.44 (1.12-1.85)** | **1.32 (1.02-1.70)** | 1.25 (0.97-1.62) | 1.08 (0.82-1.42) |
| Trajectory 5 | **2.23 (1.36-3.66)** | 1.51 (0.92-2.48) | 1.42 (0.86-2.34) | 1.32 (0.80-2.19) | 1.12 (0.67-1.87) |

Model A – Univariable

Model B – Multivariable adjustment for age and ethnicity

Model C – Model B + body mass index, physical activity level, smoking and alcohol consumption

Model D – Model C + pre-existing co-morbidities (cardiovascular disease, diabetes mellitus, cancer, asthma, chronic obstructive pulmonary disease) and serum low-density lipoprotein cholesterol

Model E – Model D + antihypertensive treatment

Median (interquartile range) follow-up was 9.4 (8.0-10.9) and 9.4 (7.8-11.0) years amongst men and women, respectively.

Statistically significant results (*P* < 0.05) are highlighted in **bold**.
